# Attitudes towards the sharing of genetic information with at-risk relatives: results of a quantitative survey

**DOI:** 10.1007/s00439-015-1612-z

**Published:** 2015-11-26

**Authors:** Timothy J. Heaton, Victoria Chico

**Affiliations:** School of Mathematics and Statistics, University of Sheffield, Hicks Building, Hounsfield Road, Sheffield, S. Yorkshire S3 7RH UK; School of Law, Bartolome House, University of Sheffield, Winter Street, Sheffield, S. Yorkshire S3 7ND UK

## Abstract

To investigate public attitudes towards receiving genetic information arising from a test on a relative, 955 University of Sheffield students and staff were surveyed using disease vignettes. Strength of attitude was measured on whether, in the event of relevant information being discovered, they, as an at-risk relative, would want to be informed, whether the at-risk relative’s interest should override proband confidentiality, and, if they had been the proband, willingness to give up confidentiality to inform such relatives. Results indicated considerably more complexity to the decision-making than simple statistical risk. Desire for information only slightly increased with risk of disease manifestation [log odds 0.05 (0.04, 0.06) per percentage point increase in manifestation risk]. Condition preventability was the primary factor increasing desire [modifiable baseline, non-preventable log odds −1.74 (−2.04, −1.44); preventable 0.64 (0.34, 0.95)]. Disease seriousness also increased desire [serious baseline, non-serious log odds −0.89 (−1.19, −0.59); fatal 0.55 (0.25, 0.86)]. Individuals with lower education levels exhibited much greater desire to be informed [GCSE log odds 1.67 (0.64, 2.66)]. Age did not affect desire. Our findings suggest that attitudes were influenced more by disease characteristics than statistical risk. Respondents generally expressed strong attitudes demonstrating that this was not an issue which people felt ambivalent about. We provide estimates of the British population in favour/against disclosure for various disease scenarios.

**Electronic supplementary material:**

The online version of this article (doi:10.1007/s00439-015-1612-z) contains supplementary material, which is available to authorized users.

## Introduction

The past decade of progress in genetics has provided transformative opportunities for human health through improved diagnosis, prevention and treatment of disease (Green et al. 2011). Knowledge of an individual’s genome allows access to personal health risks and the potential for preventative and tailored treatment or, in the case of a disease that cannot be avoided, a chance to prepare for its development (Bradbury et al. 2015; Bunnik et al. 2015; Wilde et al. 2011; Foster et al. 2009; Sanderson et al. 2004). However, genetic knowledge presents significant societal challenges because the information discovered is not solely personal. Genetic tests provide information that is not relevant just to the individual tested (proband) but also that person’s family. This raises the issue of whether such information should be shared with relatives and what should be done if the proband is unwilling to share.

Clinical genetics services have faced the issue of whether to share genetic information with at-risk relatives for decades. However, the rise of next generation sequencing in clinical care, in the form of targeted panel testing and whole genome sequencing^1^ and the mainstreaming of genetic medicine have greatly increased the prevalence of such familial disclosure issues. Furthermore, as described by Lucassen and Parker (2010), the technological developments in the possibilities for treatment have made finding the correct balance between the competing public interests of patient confidentiality and the avoidance of harm to relatives both more difficult and pertinent.

Professional guidance on when it may be permissible to share genetic information with at-risk relatives does exist. In the UK, the Joint Committee on Medical Genetics (RCP, RCPath, BSHG 2011) views as good practice “appropriate use of [a proband’s] genetic information to benefit the clinical management of family members” and recommends attempting to obtain consent for such communication from a proband prior to any genetic investigation. However, in situations where the scope of such consent is unclear or has been refused, the Joint Committee recognises that there may be circumstances where disclosure to prevent serious harm in a relative is still justified. Similar guidance is found in other genomic (Human Genetics Commission Inside information 2002) and non-genomic clinical contexts (General Medical Council Consent: patients and London: GMC 2008). In the US, the American Society of Human Genetics (American Society of Human Genetics 1998) and the President’s Commission for the Study of Ethical Issues in Biomedical and Behavioural Research (President’s Commission for the Study of Ethical Problems in Medicine and Biomedical Behavioural Research 1983) have also provided professional guidance. They too support gaining proband consent before disclosing information to relatives but also outline circumstances in which they consider it acceptable to breach patient confidentiality if such consent in refused—if serious, immediate, and foreseeable harm to relatives was likely to occur that could otherwise be prevented.

Despite this guidance, the issue of when disclosure to family members might be acceptable (or even desirable) is still highly contentious and subject to legal challenge. In the US, relatives’ desire to receive genetic information has led to a legal duty to warn relatives of familial health risks. However, it is not clear whether this duty can be discharged by informing the proband (*Pate v Threlkel*) or whether the duty prevails if the proband prefers not to disclose (*Safer v Pack*). A similar duty does not exist in the UK. Thus, UK physicians have to make difficult assessments of patients’ and relatives’ interests in knowing, not knowing and in having their confidence respected. UK hospitals have reached out of court settlements for failing to warn family members of known genetic risks discovered from tests on a relative (British Society for Genetic Medicine Annual Conference, Arena and convention centre Liverpool. private communication, September 2013). However, the English courts have recently denied that doctors owe an at-risk family member a duty to be informed in the face of the proband’s refusal to consent to the release of such information (*ABC v St George’s Healthcare NHS Trust & others*). However, the decision to grant the aggrieved relative leave to appeal in this case means that the legal position on disclosure to at-risk relatives remains unclear.

As the Joint Committee on Medical Genetics (RCP, RCPath, BSHG 2011) states, the fundamental challenge for health professionals in making decisions on disclosure to at-risk relatives is in the balancing of three competing tensions: confidentiality to patients; the potential benefit of sharing information with family members; and respecting the possibility such family members may wish not to receive such information. As a consequence it is vital that any guidance given to health professionals is seen to reflect public opinion.

There is a growing body of literature considering disclosure of unexpected genetic findings to individuals. However, much of this research concentrates on the return of results to probands rather than the question of interest here—the disclosure of information to at-risk relatives. This work on the return of genetic information to probands primarily focuses on the views of patients regarding the receipt of information themselves from their own test (e.g. Middleton et al. 2015; Clift et al. 2015; Facio et al. 2013; McGowan et al. 2013). While there are some overlaps between this issue and the disclosure to at-risk relatives there are also significant differences. Furthermore, much of this literature on return of results to probands considers the research rather than clinical context, where different concerns arise (Middleton et al. 2015). It can, therefore, only provide limited insight into questions relating to the disclosure to at-risk relatives.

Whilst there is a literature considering disclosure of genetic information to at-risk relatives, much is based upon theoretical and ethical positions (see, for example, Parker 2012; Chico 2012; Knoppers 2002; Knoppers et al. 1998). Gaff and Bylund (2010) also provide a practical framework, based upon family communication theory, on the approach to the communication and informing of family members about genetic information. Empirical work on disclosure to family members can be split into studies on the attitudes/experiences of three distinct groups: genetic health professionals, patients, and would-be relatives. A systematic review of this work can be found in Dheensa et al. (2015). Until now, the predominant focus has been on the attitudes of genetic health professionals (Klemenc-Ketiš and Peterlin 2014; Yu et al. 2014; Strong et al. 2014; Ramoni et al. 2013; Lemke et al. 2013; Stol et al. 2010; Erde et al. 2006; Clarke et al. 2005; Falk et al. 2003; Dugan et al. 2003). There are a small number of primary studies considering *patients’* attitudes to disclosure of their test results to relatives (Kohut et al. 2007; Pentz et al. 2005; Wilcke et al. 1999). However, there are very few empirical studies which investigate *unsuspecting relatives, or potential unsuspecting relatives’* views regarding what information they do, or do not, want to receive (Daack-Hirsch et al. 2013; Wolff et al. 2007; Suthers et al. 2006). Amongst these three distinct study groups, health professionals generally express a feeling of responsibility towards at-risk relatives but identify difficulties in acting upon this responsibility. The views of the public appear more varied although the limited studies on would-be relatives indicate that the majority of people do want to be informed about the existence of a hereditary disease within their family and consider breaches of proband confidentiality acceptable in certain circumstances.

This paper begins to address the need to gather and understand public opinion and investigates whether the current recommendations reflect public views. We investigate, via a survey of university staff and students, attitudes on disclosure of unsolicited genetic information to at-risk relatives following the testing of another individual. We quantify the specific factors influencing strength of attitude on what information at-risk relatives wish to know; whether an at-risk relatives’ interest should override any views of the proband; and willingness to forgo one’s own confidentiality. We study the effect of both the characteristics of the disease such as seriousness, preventability and risk of it manifesting; and also the personal demographics of the respondent (e.g. age and sex). In an extension, we consider what the views of our university-based respondents suggest about the wider population. Reweighting our sample to reflect the demographics of the British (i.e. English, Scottish and Welsh) population, we provide preliminary estimates on the proportion of the public who do, and do not, favour disclosure together with what information they do, and do not, want to know.

## Materials and methods

### Data collection and study design

#### Questions of interest

**Fig. 1:**
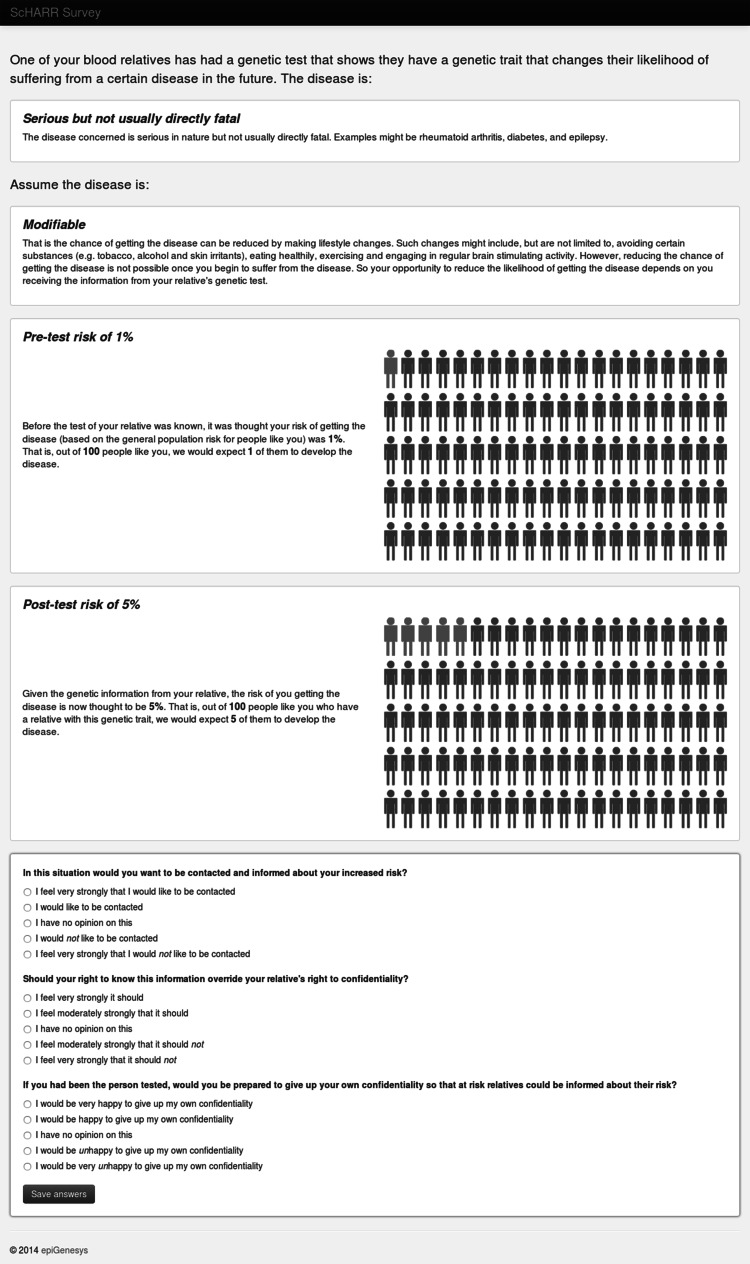
A sample vignette shown to respondents. Numerical risks were visually represented as well as given in the text. Examples were also given as to what was meant by the differing seriousness and modifiability disease categories to increase comparability between respondents

We analysed opinion on three specific questions relating to the disclosure of genetic information to at-risk relatives, see Fig. 1. For a particular genetic finding on a proband (1) would an at-risk relative wish, or not wish, to be contacted and informed about their resultant risk of a disease, (2) whether these at-risk relatives believed their interest should override the proband’s confidentiality, and (3) if the relative themselves had been the tested individual, how willing did they feel they would be to give up their own confidentiality so that at-risk relatives could be informed?

To investigate the factors influencing attitudes on these three questions, we created an online survey presenting a set of vignettes showing a range of possible disease scenarios. The disease characteristics chosen to vary between vignettes were informed by preliminary focus groups.

#### Preliminary focus groups

We ran two focus groups to determine the potential key factors affecting desire to receive genetic information which could then be varied in the vignettes and tested for their influence on attitudes. Focus group participants were recruited via a public engagement event at the University of Sheffield and a local newspaper. Sixteen females and 8 males, with ages ranging from early 20s to late 70s and covering a mix of employed, unemployed and retirees from manual, skilled and professional occupations attended. Groups were audio-recorded before being transcribed and inputted to NVivo (Version9). Analysis identified and categorised topics and frequencies before establishing primary, secondary and lower level coding for the factors influencing the group’s views, priorities and preferences.

#### Vignette design and online survey

The seriousness of the condition, the absolute risks of disease manifestation for the at-risk relative both before and after the test on the proband was performed, and the possibility of disease prevention were identified as the potential key influences to be tested by variation between vignettes. Details on the levels of each factor and overall design can be seen in Table 1. This provided us with a vignette bank of 54 differing disease scenarios. Each participant in the online survey was presented with four vignettes selected uniformly at random from this bank and asked for their attitudes on our three questions of interest. Visual representations of the absolute numerical risks, as recommended by the Presidential Committee for the Study of Bioethical Issues (Presidential Commission for the Study of Bioethical Issues 2013), were used alongside written explanations to aid comprehension (see Fig. 1 for a sample vignette). The draft survey was piloted with three individuals recruited from the focus groups to determine whether the questions were understood as intended. Ethical approval was obtained from the University of Sheffield Research Ethics Committee.

**Table 1:**
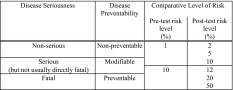
Levels of factors chosen for quantitative survey

We defined three hypothetical levels for the disease seriousness and three for preventability. We also selected two levels of pre-test risk for the at-risk relative, each with a further three levels of post-test risk. To create a vignette, one level was chosen from each factor at random

#### Outcome measures

Responses to our three questions of interest were measured on a five-point Likert-type scale to rate strength of attitude ranging from strong no/strongly disagree (response 1) through no opinion (response 3) to strong yes/strongly agree (response 5). Personal demographics, also based on themes identified by the focus groups, were collected for each respondent (five characteristics). It was made explicitly clear that respondents should base their decisions solely upon their personal desire for the information and to assume it would not also be disclosed to third parties, for example, insurance companies.

#### Participants

The survey was made available to the University of Sheffield volunteers list^2^ for a period of three months from October to December 2013. During this time responses from 955 students and staff were obtained. This provided views on 3820 scenarios. See Table S1 in supplementary information for demographic details on respondents.

### Analysis

#### Proportional odds logistic regression

To investigate the factors influencing attitudes, a proportional odds logistic regression with random effects was fitted to the responses using Bayesian MCMC. Further details can be found in Appendix A. Baseline views correspond to a 25–40-year-old female who has a university education, is not religious and does not have a partner. The baseline disease is serious and modifiable; pre- and post-test baseline risks are taken to be zero.

#### Extension to the wider population—reweighting demographics

While our study was performed on university-based respondents and, being an online survey, permitted self-selection amongst respondents, we can still consider what it might tell us about the views of the wider population. This can be achieved by reweighting, according to the demographic information, the responses of our survey to represent the population as a whole. NatCen Social Research British social attitudes survey 2nd Edition (2011) provides a breakdown of the proportion of individuals of each age, sex, education level, relationship status and religious view. For each of these groups, our proportional odds logistic model can provide an estimate of views. These can then be combined to provide an estimate of a representative sample of society. To provide such estimates, 2000 samples were drawn from our MCMC chains for each of our three questions of interest. We report the means and 95 % intervals for the predicted proportion of individuals in the British population holding each view.

## Results

Consistent values were reflected across the three questions. A stronger desire to know information as a prospective at-risk individual usually corresponds to a stronger belief that the relative’s right to know should override the proband’s interest in confidentiality (Kendall correlation 0.51, *p* value <2 × 10^−16^). It also corresponds to a greater happiness to forgo one’s own confidentiality to inform at-risk relatives (0.36, *p* value <2 × 10^−16^).

Tables 2, 3 and 4 provide posterior estimates and 95 % credible intervals for the effect of the various disease and personal characteristics on our three questions of interest. Positive values indicate individuals/diseases with the particular characteristic are more likely to give a higher (more positive) response; conversely negative values are more likely to lead to a lower (more negative) response. The estimates of random effect size quantify the amount of natural variation amongst the population.

**Table 2:** Factors affecting an individual’s desire to know their genetic information

Category	Posterior	Quantiles	
	Mean	2.5 %	97.5 %
**Age**			
16–25	0.08	−0.23	0.41
25–40	–	–	–
40–60	−0.18	−0.62	0.25
Over 60	−0.55	−1.46	0.39
**Max. education level**			
GCSE	1.67	0.64	2.66
A-level	0.09	−0.31	0.50
University	–	–	–
**Other demographic factors**			
In relationship	−0.07	−0.38	0.26
Religious	−0.14	−0.45	0.16
Sex (male)	0.26	−0.01	0.54
**Type of disease**			
*Non-serious*			
Non-preventable	−2.44	−2.73	−2.14
Modifiable	−0.89	−1.19	−0.59
Preventable	−0.61	−0.90	−0.31
*Serious (but not fatal)*			
Non-preventable	−1.74	−2.04	−1.44
Modifiable	–	–	–
Preventable	0.64	0.34	0.95
*Fatal*			
Non-preventable	−1.73	−2.03	−1.43
Modifiable	0.55	0.25	0.86
Preventable	1.23	0.91	1.54
**Risk factors**			
Pre-risk	−0.07	−0.09	−0.05
Post-risk	0.05	0.04	0.06
**Random effect** (Population variation)			
σ	1.74	1.60	1.89
**Category boundaries** (desire to be contacted)			
V. strong no/no, * a*_1_	−4.71	−5.11	−4.29
No/no opinion, *a*_2_	−2.55	−2.90	−2.17
No opinion/yes, *a*_3_	−1.71	−2.06	−1.35
Yes/V. strong yes, * a*_4_	1.00	0.66	1.35

**Table 3:** Factors affecting an individual’s belief that their right to know, as an at-risk relative, should override the confidentiality of the proband

Category	Posterior	Quantiles	
	Mean	2.5 %	97.5 %
**Age**			
16–25	0.18	−0.26	0.61
25–40	–	–	–
40–60	0.09	−0.48	0.70
Over 60	−0.40	−1.67	0.83
**Max. education level**			
GCSE	1.99	0.69	3.30
A-level	−0.07	−0.64	0.49
University	–	–	–
**Other demographic factors**			
In relationship	−0.20	−0.65	0.24
Religious	0.02	−0.41	0.43
Sex (male)	−0.03	−0.43	0.38
**Type of disease**			
*Non-serious*			
Non-preventable	−2.23	−2.54	−1.92
Modifiable	−0.86	−1.17	−0.55
Preventable	−0.44	−0.75	−0.14
*Serious (but not fatal)*			
Non-preventable	−1.07	−1.38	−0.77
Modifiable	–	–	–
Preventable	0.60	0.30	0.91
*Fatal*			
Non-preventable	−0.81	−1.13	−0.49
Modifiable	0.84	0.53	1.15
Preventable	1.54	1.22	1.86
**Risk factors**			
Pre-risk	−0.05	−0.07	−0.03
Post-risk	0.04	0.03	0.04
**Random effect** (Population variation)			
σ	2.68	2.50	2.87
**Category boundaries** (should right to know override confidentiality?)			
Strong no/no, *a*_1_	−3.37	−3.86	−2.88
No/no opinion, *a*_2_	−0.52	−0.99	−0.06
No opinion/yes, *a*_3_	0.30	−0.17	0.75
Yes/strong yes, *a*_4_	3.13	2.63	3.60

**Table 4:** Factors affecting whether an individual would be happy to forgo their right to confidentiality in the event they were a proband in a genetic test from which information, pertaining to a disease was found that was of relevance to a relative

Category	Posterior	Quantiles	
	Mean	2.5 %	97.5 %
**Age**			
16–25	0.27	−0.44	0.89
25–40	–	–	–
40–60	−0.06	−0.87	0.69
Over 60	0.86	−0.79	2.54
**Max. education level**			
GCSE	1.57	−0.17	3.29
A-level	0.26	−0.46	0.95
University	–	–	–
**Other demographic factors**			
In relationship	0.59	0.01	1.19
Religious	−0.73	−1.25	−0.20
Sex (male)	−0.20	−0.67	0.27
**Type of disease**			
*Non-serious*			
Non-preventable	−1.86	−2.24	−1.47
Modifiable	−0.79	−1.18	−0.40
Preventable	−0.39	−0.78	0.00
*Serious (but not fatal)*			
Non-preventable	−1.12	−1.51	−0.74
Modifiable	–	–	–
Preventable	0.54	0.14	0.94
*Fatal*			
Non-preventable	−1.00	−1.39	−0.60
Modifiable	0.33	−0.06	0.73
Preventable	1.00	0.59	1.42
**Risk factors**			
Pre-risk	−0.07	−0.10	−0.05
Post-risk	0.05	0.04	0.06
**Random effect** (Population variation)			
σ	3.12	2.87	3.39
**Category boundaries** (as the proband would you forgo confidentiality?)			
V. unhappy/unhappy, *a*_1_	−7.28	−8.18	−6.42
Unhappy/no opinion, *a*_2_	−4.88	−5.70	−4.07
No opinion/happy, *a*_3_	−3.99	−4.79	−3.18
Happy/V. happy, * a*_4_	−0.47	−1.24	0.31

We also present in Table 5, and Table S2 in supplementary information, our estimates (based on survey reweighting) of the proportion of the British population in each response category together with 95 % intervals for each combination of disease seriousness and preventability. Since the absolute changes affected by pre- and post-test risk are small we only provide two illustrative levels of pre- and post-test risk. Table 5 (used for discussion below) presents the views for diseases where the pre-test risk for the concerned relative was believed to be 1 % and the post-test risk 2 % and Table S2 presents the views for a pre-test risk of 10 % and a post-test risk of 20 %. In all three questions asked, few individuals fall into the class of “no opinion”, indicating strong attitudes and little ambivalence regarding disclosure and receipt of information on genetic risks.

**Table 5:** Estimated views of representative sample of British public for different disease categories when pre-risk is 1 % and post-risk is 2 %

	Non-preventable				Modifiable				Preventable			
	S. no	≤No	≥Yes	S. yes	S. no	≤No	≥Yes	S. yes	S. no	≤No	≥Yes	S. yes
Would you want to know this information?												
Non-serious	13.5 (10, 18)	38.9 (32, 47)	48.4 (40, 57)	14.4 (9, 21)	4.7 (3, 7)	19.2 (15, 25)	70.9 (63, 77)	31.5 (23, 40)	3.8 (3, 6)	16.4 (12, 22)	74.5 (68, 80)	35.4 (27, 44)
Serious	8.6 (6, 12)	29.2 (23, 36)	59 (51, 66)	21.1 (14, 29)	2.3 (2, 4)	11.4 (8, 16)	81.4 (76, 86)	44.4 (35, 53)	1.3 (1, 2)	7.4 (5, 10)	87.1 (83, 91)	54.1 (45, 62)
Fatal	8.6 (6, 12)	29.1 (23, 36)	59.1 (51, 67)	21.2 (15, 29)	1.5 (1, 2)	7.9 (6, 11)	86.4 (82, 90)	52.7 (44, 61)	0.8 (0, 1)	4.9 (3, 7)	91.1 (88, 94)	62.8 (54, 70)
Should sharing override confidentiality of testee?												
Non-serious	28.7 (23, 36)	60.8 (52, 70)	30.4 (22, 39)	9.2 (5, 15)	16.8 (12, 22)	45 (37, 54)	45.5 (36, 54)	17.5 (11, 25)	13.9 (10, 19)	40.1 (33, 49)	50.4 (42, 59)	20.8 (14, 29)
Serious	18.4 (13, 24)	47.4 (39, 56)	43.1 (34, 52)	16 (10, 23)	11.2 (8, 15)	35.3 (28, 44)	55.5 (46, 64)	24.6 (17, 33)	8.2 (6, 12)	28.9 (23, 36)	62.4 (54, 70)	30.5 (22, 39)
Fatal	16.4 (12, 22)	44.3 (36, 53)	46.1 (37, 55)	17.9 (12, 25)	7.3 (5, 10)	26.6 (21, 34)	64.9 (57, 72)	32.9 (25, 42)	4.8 (3, 7)	20.3 (15, 27)	72.2 (65, 79)	40.6 (32, 50)
Would you be willing to forgo your confidentiality if you were tested?												
Non-serious	5.2 (3, 8)	16.1 (11, 22)	77.5 (70, 84)	43.3 (33, 54)	2.9 (2, 5)	10.2 (7, 15)	85 (79, 90)	54.5 (44, 64)	2.3 (1, 4)	8.4 (5, 13)	87.3 (82, 91)	58.6 (48, 68)
Serious	3.5 (2, 6)	11.8 (8, 17)	82.9 (76, 88)	51 (40, 61)	1.8 (1, 3)	7 (5, 10)	89.3 (85, 93)	62.5 (53, 71)	1.3 (1, 2)	5.3 (3, 8)	91.7 (88, 95)	67.7 (58, 76)
Fatal	3.2 (2, 5)	11.2 (8, 16)	83.7 (77, 89)	52.3 (42, 62)	1.4 (1, 2)	5.9 (4, 9)	90.9 (87, 94)	65.9 (56, 74)	0.9 (0, 2)	4.1 (2, 6)	93.4 (90, 96)	72 (63, 79)

Estimates of those expressing “no opinion” can be found by calculating 100 − (proportion ≤ no) − (proportion ≥ yes)

### What information do at-risk relatives want to know?

#### Factors affecting attitude

As shown in Table 2, the most critical factor affecting desire to be informed is whether action can be taken to avoid the disease. As indicated in the increase in $\widehat{{\gamma_{k} }}$ from non-preventable through modifiable to preventable, such desire increases with preventability, e.g. compared with modifiable baseline, non-preventable log odds of −1.74 (−2.04 to −1.44) and preventable log odds 0.64 (0.34–0.95) for a serious but non-fatal condition. Individuals are also more likely to want information as the condition becomes more serious, e.g. considering a modifiable condition we have, compared to the serious disease baseline, a non-serious disease log odds of −0.89 (95 % CI −1.19 to −0.59) and a fatal disease log odds of 0.55 (95 % CI 0.25–0.86). Altering either of these characteristics has large absolute effects on strength of opinion. Desire also increases as your believed risk of developing the disease, in light of the test, increases (log odds 0.05, with 95 % CI of 0.04–0.06, for each 1 % increase in posterior risk). Individuals are less concerned to know about diseases which are already more common in the population (pre-risk log odds −0.07 with 95 % CI of −0.09 to −0.05). However, while these two risk factors are statistically significant their absolute effect on attitude is small compared with the other disease characteristics. This suggests that either individuals struggle to fully understand quantitative risk in the context of genetic information, or that decisions are primarily made based upon attitudes towards the possession of personalised (and familial) information relating to oneself per se rather than actual informativeness.

Age does not have a significant influence (95 % CIs for all age groups overlapping 0). Neither does relationship status (95 % CI of −0.38 to 0.26) nor religious belief (95 % CI of −0.45 to 0.16). There is strong evidence that those individuals with GCSE or equivalent as their highest level of qualification are considerably more likely to desire information about themselves ($\hat{\beta }$ = 1.67, 95 % CI of 0.64–2.66) while little difference is seen between those educated to A-level or beyond. There is also some evidence that men have a stronger desire for information than women (0.26, 95 % CI −0.01 to 0.54).

#### Proportions in the general population

For conditions which are either modifiable or preventable, our survey suggests that (were the views of our university respondents to be reflected across wider society) a significant majority of the public would want to be informed. For a preventable and fatal disease, our survey predicts 91 % (CI 88–94 %) “would like to be contacted” and 63 % (CI 54–70 %) would feel “very strongly that [they] would like to be contacted”. This desire to be informed also extends to non-serious conditions. Few individuals would appear to feel, for a modifiable or preventable condition, “that [they] would NOT like to be contacted”; modifiable and non-serious 19 %, CI 15–25 %; preventable and fatal 5 %, CI 3–7 % with very few feeling “very strongly that [they] would NOT like to be contacted”. This perhaps suggests that in the case of a modifiable or preventable disease, we should not be too concerned about giving information that people might not want to know because very few people have such a desire. For non-preventable conditions, however, our survey suggests that approximately 25–40 % may not want to receive genetic information about themselves no matter what the level of seriousness—fatal 29 % (23–36 %); serious 39 % (32–47 %); and non-serious 39 % (32–47 %).

### Should an at-risk relative’s right to know override proband confidentiality?

#### Factors affecting attitude

Table 3 demonstrates that excepting those with a maximum education level of GCSE who are more likely to believe disclosure should take precedence ($\hat{\beta }$ = 1.99, 95 % CI 0.69–3.30), personal characteristics make no significant difference to views on the relative importance of maintaining confidentiality. Potential for disease prevention again has the largest impact with support for overriding confidentiality increasing with preventability, e.g. compared to modifiable baseline, non-preventable log odds −1.07 (−1.38 to −0.77) and preventable 0.60 (0.30–0.91). Disease seriousness has an almost equally significant role with support for disclosure increasing with seriousness (non-serious −0.86, −1.17 to −0.55; fatal 0.84, 0.53–1.15). Pre-test risk (−0.05, 95 % CI −0.07 to −0.03) and post-test risk (0.04, 95 % CI 0.03–0.04) of disease development are shown to be statistically significant factors but again the absolute change in opinion they affect is small in comparison to changes in disease seriousness and preventability.

#### Proportional views in the general population

Where the condition is non-preventable, demographic reweighting of our survey responses suggests that the majority of the public do not believe their right to know should override the proband’s right to confidentiality (non-serious 61 %, 52–70 %; fatal 44 %, 36–53 %). The proportion who feel strongly that [their] right to know should NOT override the proband’s right to confidentiality is high (non-serious 29 %, 23–36 %; fatal 16 %, 12–22 %). However, where the disease is either serious or fatal, and some level of preventative action is possible, support for overriding proband confidentiality increases greatly. In the case of a fatal and preventable disease, our survey suggests only 20 % (15–27 %) of the public believe their right to know should not override the proband’s confidentiality, compared with 72 % (65–79 %) who believe it should and 40 % (CI 32–50 %) strongly believing so.

### Are individuals willing to forgo their own confidentiality in genetic tests so that an at-risk relative could be informed?

#### Factors affecting attitude

Table 4 indicates increased willingness to forgo confidentiality for diseases which are more preventable (compared to baseline, non-preventable −1.12, −1.51 to −0.74; preventable 0.54, 0.14–0.94) and serious (non-serious log odds −0.79, 95 % CI −1.18 to −0.40; fatal 0.33, −0.06 to 0.73). Of these two factors, preventability is again predominant. Pre-test (−0.07, −0.10 to −0.05) and post-test (0.05, 0.04–0.06) disease risks also have a statistically significant effect but small in absolute terms. There is some evidence that those in relationships are happier to forgo confidentiality (0.59, 0.01–1.19) while those who are religious are less willing (−0.73, −1.25 to −0.20). There is no evidence that age, sex or education level significantly affect willingness to forgo one’s own confidentiality.

The random effect standard deviation (3.12, 2.87–3.39) is large indicating this question has the largest spread of views amongst the population. Despite a general willingness to share, some individuals are strongly against the idea of forgoing their own confidentiality whatever the information. 20 of our 955 participants indicated an unhappiness to forgo confidentiality in all vignettes presented. Interestingly, these particular 20 individuals were not consistent in their views across the alternate questions with a significant number responding that, were they the at-risk relative, they would want to be told. Some also felt that such disclosure was more important than proband confidentiality.

### Proportional views in the general population

Irrespective of the nature of the information, people appear generally happy to forgo their own confidentiality in the context of genetic findings relevant to family members. In the case of a fatal and preventable disease, we estimate 93 % (90–96 %) of the British public would be willing to forgo their confidentiality with 72 % (63–79 %) strongly so. For a non-serious and non-preventable disease these proportions drop to 77 % (70–84 %) and 43 % (33–54 %), respectively. A small proportion (1 % for a fatal and preventable disease) is indicated to be unwilling to forgo their confidentiality in any circumstance.

## Discussion

### What does this study tell us?

Our study of 955 university-based respondents shows that making decisions about disclosing and receiving genetic information in families is extremely complex and based on much more than statistical risk. We found that the most important factor affecting an at-risk relative’s desire to know genetic information is the preventability of the disease to which the information relates. Disease seriousness is also highly important. While informativeness of the test, as measured by the increase in the believed risk of disease development for the at-risk relative from pre- to post-test, is seen to have a statistically significant the absolute effect is small in comparison to the disease seriousness or preventability. This suggests that even when a test indicates in a small increase in the chance of developing a disease, at-risk relatives may still want to be informed if action is possible to modify this risk of onset. Such views may be due to decisions being based upon attitudes towards possession of any available information about oneself per se, the familial nature of the information, or perhaps simply a difficulty in understanding quantitative genetic risk. Views were mainly unaffected by demographic factors although those with lower levels of education express significantly more desire to be informed than those with higher level qualifications (above GCSE). The values that affect an individual’s desire for information have a largely consistent effect on whether an individual believes the right of the at-risk relative should override the proband’s confidentiality and also their happiness to forgo their own confidentiality in the event they themselves were tested.

The Joint Committee on Medical Genetics (RCP, RCPath, BSHG 2011) reports the “feelings of altruism and solidarity towards family members” that patients experience and notes that the majority are happy for their information to be shared. This position is reflected in our empirical work. Where patients are not happy to share information, professional guidance (RCP, RCPath, BSHG 2011; HCG 2002; American Society of Human Genetics (1998); President’s Commission for the Study of Ethical Problems in Medicine and Biomedical Behavioural Research 1983) reflects a consensus that there are certain circumstances where breach of patient confidentiality to inform relatives can be justified. Where confidentiality would be breached, much of this guidance only considers disclosure permissible when there is a high probability that serious harm will occur to the at-risk relative which could otherwise be avoided. This position to permit disclosure only in exceptional circumstances is not fully reflected in the views of our respondents. Since attitudes do not appear to be made primarily on the basis of statistical risk, the likelihood of harm does not appear to be the overriding factor influencing attitudes regarding when a breach of proband confidentiality is justified.

### Strengths, weaknesses and further study

We believe our study to be the first large-scale (955 respondents) quantitative study into the factors affecting attitudes to the disclosure of genetic information to unsuspecting at-risk relatives that specifically surveys the views of potential relatives through a selection of the population. The highly visual vignettes and simplicity of the design allowed scenarios to be easily understood and meant the survey maintained enthusiasm of respondents. We believe this, together with the clear interest in this issue amongst the public, can be seen by the large number of responses obtained.

Our measurement of strength of attitude is important in weighing the competing interests of proband and relative to inform public attitudes to the disclosure of genetic information. Use of multiple responses also enables assessment of the variation in opinion amongst the population. Quantifying the impact of the absolute risks of disease manifestation may also help inform how far disclosure should extend within a family. Finally, via a sample reweighting, we are able to consider how our survey responses may generalise to the wider population providing preliminary estimates of the views of the British public.

Our study has limitations. Respondents were drawn from university staff and students who may have different views from those outside a university environment. In addition there is potential for self-selection bias within our sample. The survey was freely available online possibly leading to more responses from individuals with polarised views. Our extension estimating the proportion of the British population holding each view should, therefore, be treated as preliminary. Further work is needed to recruit a truly representative sample.

Our survey is also only able to ask people about their attitudes in hypothetical circumstances. How accurately hypothetical views match behaviour in the context of a real situation is a potential criticism of our work. While much research has demonstrated a significant and substantial attitude–behaviour link (Glasman and Albarracin 2006, Kraus 1995) there are several cases where the uptake of genetic testing in practice has been considerably lower than self-reported interest would have suggested (e.g. Binedell and Soldan, 1997). It is not evident that this will affect the relative importance of the various factors in decision-making but it is possible that the real desire for genetic information may be below that reported in our study. This raises an important further issue since discovering whether or not a potential at-risk relative wants to know a risk that is, as yet, unknown to them will always require a hypothetical approach. If one wishes to respect such a relative’s autonomy it is important to make sure that these self-reported hypothetical attitudes do agree with desired behaviour in reality. Glasman and Albarracin (2006) show that attitudes and behaviour are most closely aligned when views are held strongly, are stable over time and are based upon direct experience. While several of our scenarios demonstrate that individuals do have strong views, much of the public is likely to have limited direct experience of genetic medicine. It is, therefore, crucial that individuals are fully educated before they make such decisions.

Additionally, we only presented disease categories (e.g. serious, modifiable, …). Views on the nature of these categories may vary amongst respondents and health professionals (Wertz and Knoppers 2002). A potential extension would be to study opinion for a range of specific disease states; for example, those recommended for screening by the American College of Medical Genetics and Genomics (Green et al. 2013) and Genomics England.^3^ Individuals may, however, have limited/varying knowledge about such named conditions.

Our survey did not collect information on the professional background of the respondents. It would be of interest to investigate whether, in the presented vignettes, the attitudes of healthcare professionals differed from those of the general public. Middleton et al. (2015) have recently demonstrated that there is a disconnect between the views of those handling research findings and those participating in research with regard to the feedback of genetic information. Work to investigate the reasons behind the observed differences in attitudes amongst those with lower education would be valuable.

### Implications for research and practice

Studying the views of the public towards disclosing genetic information to family members adds a valuable perspective to the existing empirical literature on professional views (Dheensa et al. 2015; Klemenc-Ketiš and Peterlin 2014; Lemke et al. 2013; Stol et al. 2010; Falk et al. 2003; Dugan et al. 2003; Clarke et al. 2005, Yu et al. 2014; Strong et al. 2014; Ramoni et al. 2013; Erde et al. 2006). Our work demonstrates people are not ambivalent about sharing genetic information within families. Instead, people have strong attitudes. This work further shows that attitudes to receiving and sharing genetic information in families might not rest predominantly on the particular characteristics of the information itself, but may instead be based on the individual’s attitude more generally to having information about themselves which exists and is known by others. The complex and nuanced picture of attitudes to receiving unsolicited genetic information demonstrated here complicate any effort to produce a single set of guidelines to inform when disclosure to at-risk family members should occur.

The ability to modify the risk of manifestation does, however, appear to be a key criterion perhaps with individuals coping more easily with adverse information if they can act practically upon it. For diseases which are modifiable in some way our survey suggests a strong desire to be informed. In such circumstances, clinicians may wish to favour disclosure where this does not breach confidence because causing grievance based on a desire not to know is unlikely given the strong attitudes towards desiring actionable information shown in this survey.

Medical actionability is not the only important factor. Desire for information also increases with disease seriousness and is significant even when the condition is not preventable suggesting individuals value being able to plan lifestyle and welfare issues in the light of knowledge they are at an increased risk of suffering from a serious condition (Foster et al. 2009; Bunnik et al. 2015; Bradbury et al. 2015). Desire to know in such circumstances is, however, balanced by a stronger desire not to know amongst some individuals. Here, therefore, there may be a risk of grievance if people are informed when they feel they would have preferred not to know.

Our work also suggests people are highly willing to share personal information with members of their family. While the sharing of health data outside of the NHS is currently highly contentious, as demonstrated by ABC v St George’s Healthcare NHS Trust and others and Care.data,^4^ it might be that concerns about confidentiality of health information are not common place in familial relationships and patients are happy to adopt a familial model to the sharing of genetic information (d’Agincourt-Canning 2006). Although there seems to be a small minority who would not be happy to share their information it might be that a cultural shift where sharing becomes the norm will influence behaviour (see Bicchieri and Chavez (2010); Bicchieri (2009) for the effect of social norms on behaviour). Over time, the minority who oppose sharing may then find their position difficult to maintain and justify within a culture of sharing.

## Conclusion

Our results indicate disease preventability and seriousness are the key factors in determining people’s attitudes towards receiving and sharing genetic information. The actual increase in risk of disease manifestation plays a much lesser role. Most respondents reported a willingness to consent to sharing pertinent genetic information with relatives and many would want such information to be shared with them even if this was against their tested relative’s wishes. Current professional guidance recognises that breach of proband confidence might be appropriate in narrowly defined circumstances where relatives are likely to suffer serious and preventable harm. However, our work indicates that people consider breaches may be permissible in a wider, and less strict, range of circumstances.

Further work is needed on the longer term impact on individuals receiving personal and uncertain genomic information. Is receipt of such information a net good, can individuals understand the information they receive, and can it be assimilated beneficially into their lives?

## Cases

- *ABC v St George’s Healthcare NHS Trust* [2015] EWHC 1394 (QB).
- *Pate v Threlkel* “661 So. 2d 278,” 1995.
- *Safer v Pack* “291 NJ Super. 619,” 1996. NJ Super. Ct. App. Div.

## Electronic supplementary material

Supplementary material 1 (DOCX 70 kb)

## Footnotes

1 (e.g. http://www.genomicsengland.co.uk/).

2 This list comprises current and retired staff of all grades and current students at the University of Sheffield unless they have chosen to opt out and remove themselves from it.

3 http://www.genomicsengland.co.uk/library-and-resources/.

4 Care.data is an NHS England initiative to take data from GP records and upload them to the national Health and Social Care Information Centre (HSCIC) databases. The initiative had to be postponed in February 2014 due to a public backlash over, among other things, public concerns that patient information would be made available outside of the NHS, potentially to private companies.
