# Supplementary material for: Attitudes towards the sharing of genetic information with at-risk relatives: results of a quantitative survey
Source: Hum Genet. 2015 Nov 26;135:109–20. doi: 10.1007/s00439-015-1612-z (PMC4698294; doi:10.1007/s00439-015-1612-z)
Supplement: Supplementary file 1 — Supplementary material 1 (DOCX 70 kb) [file 439_2015_1612_MOESM1_ESM.docx]

## Supplementary Information

## SI1 Demographics of Survey Respondents

In Table S2 we present the demographic characteristics of our 955 survey respondents.

|  |  | Number of Participants | Percentage of Total |
| --- | --- | --- | --- |
|  |  | (n) | (% ) |
| Total | | 955 | — |
| Age | |  |  |
|  | 0–25 | 544 | 57 |
|  | 25–40 | 264 | 28 |
|  | 40–60 | 131 | 14 |
|  | 60+ | 16 | 2 |
| Sex | |  |  |
|  | Male | 333 | 35 |
|  | Female | 622 | 65 |
| Education | |  |  |
|  | GCSE | 20 | 2 |
|  | A-level | 115 | 12 |
|  | University | 820 | 86 |
| Relationship Status | |  |  |
|  | Single | 591 | 62 |
|  | Partner | 364 | 38 |
| Religious Beliefs | |  |  |
|  | Yes | 215 | 23 |
|  | No | 740 | 77 |

Table S1: *The demographic characteristics of our survey respondents*

## SI2 Estimates of views in the general population

In Table S3 we present (based on survey reweighting by demographic) estimates for the proportion of individuals in the general population lying in each response category for a disease where, for the ralative in question, the believed risk before the test was 10% and, in light of the test, this was now believed to be increased to 20%

| Would you want to know this information? | | | | | | | | | | | | |
| --- | --- | --- | --- | --- | --- | --- | --- | --- | --- | --- | --- | --- |
|  | Non-preventable | | | | Modifiable | | | | Preventable | | | |
|  | S. No | ≤ No | ≥ Yes | S. Yes | S. No | ≤ No | ≥ Yes | S. Yes | S. No | ≤ No | ≥ Yes | S. Yes |
| Non-serious | 11.3 | 34.7 | 52.9 | 17 | 3.8 | 16.4 | 74.6 | 35.5 | 3 | 13.9 | 77.9 | 39.5 |
|  | (8,15) | (28,43) | (44,61) | (11,24) | (3,5) | (12,22) | (68,80) | (27,44) | (2,4) | (10,18) | (72,83) | (31,48) |
| Serious | 7.1 | 25.5 | 63.2 | 24.4 | 1.8 | 9.4 | 84.2 | 48.8 | 1 | 6.1 | 89.3 | 58.5 |
|  | (5,10) | (20,32) | (55,70) | (17,32) | (1,3) | (7,13) | (79,88) | (39,57) | (1,2) | (4,8) | (85,92) | (50,66) |
| Fatal | 7 | 25.4 | 63.4 | 24.5 | 1.1 | 6.5 | 88.6 | 57.1 | 0.6 | 3.9 | 92.7 | 66.9 |
|  | (5,10) | (20,32) | (55,70) | (17,33) | (1,2) | (4,9) | (85,92) | (48,65) | (0,1) | (3,5) | (90,95) | (59,74) |
|  | | | | | | | | | | | | |
| Should sharing override confidentiality of testee? | | | | | | | | | | | | |
|  | Non-preventable | | | | Modifiable | | | | Preventable | | | |
|  | S. No | ≤ No | ≥ Yes | S. Yes | S. No | ≤ No | ≥ Yes | S. Yes | S. No | ≤ No | ≥ Yes | S. Yes |
| Non-serious | 26.4 | 58.1 | 32.9 | 10.4 | 15.1 | 42.2 | 48.3 | 19.3 | 12.4 | 37.4 | 53.2 | 22.8 |
|  | (21,34) | (49,67) | (24,42) | (6,16) | (11,20) | (34,51) | (39,57) | (13,27) | (9,17) | (30,46) | (44,62) | (16,31) |
| Serious | 16.6 | 44.6 | 45.9 | 17.7 | 10 | 32.7 | 58.3 | 26.9 | 7.2 | 26.6 | 65 | 33 |
|  | (12,22) | (36,54) | (37,55) | (12,26) | (7,14) | (26,41) | (49,66) | (19,36) | (5,10) | (21,34) | (57,72) | (24,42) |
| Fatal | 14.7 | 41.6 | 48.9 | 19.8 | 6.3 | 24.4 | 67.5 | 35.4 | 4.2 | 18.4 | 74.5 | 43.3 |
|  | (11,20) | (34,50) | (40,58) | (13,28) | (4,9) | (19,31) | (60,74) | (27,45) | (3,6) | (14,24) | (67,81) | (34,53) |
|  | | | | | | | | | | | | |
| Would you be willing to forgo your confidentiality if you were tested? | | | | | | | | | | | | |
|  | Non-preventable | | | | Modifiable | | | | Preventable | | | |
|  | S. No | ≤ No | ≥ Yes | S. Yes | S. No | ≤ No | ≥ Yes | S. Yes | S. No | ≤ No | ≥ Yes | S. Yes |
| Non-serious | 4.7 | 14.7 | 79.2 | 45.6 | 2.5 | 9.2 | 86.3 | 56.7 | 2 | 7.6 | 88.5 | 60.8 |
|  | (3,7) | (10,21) | (72,85) | (35,56) | (1,4) | (6,14) | (80,91) | (47,66) | (1,3) | (5,12) | (83,92) | (51,70) |
| Serious | 3.1 | 10.7 | 84.3 | 53.3 | 1.5 | 6.2 | 90.4 | 64.7 | 1.1 | 4.7 | 92.5 | 69.7 |
|  | (2,5) | (7,16) | (78,89) | (43,63) | (1,3) | (4,10) | (86,94) | (55,73) | (1,2) | (3,7) | (89,95) | (61,78) |
| Fatal | 2.9 | 10.1 | 85.1 | 54.6 | 1.2 | 5.2 | 91.8 | 68 | 0.8 | 3.6 | 94.1 | 73.9 |
|  | (2,5) | (7,15) | (79,90) | (44,64) | (1,2) | (3,8) | (88,95) | (59,76) | (0,1) | (2,6) | (91,96) | (65,81) |

Table S2: *Table showing estimated views of representative sample of British public for different disease categories when pre-risk is 10% and post-risk is 20%. Estimates of those expressing “no opinion” can be found by calculating 100 - (Proportion ≤ No) - (Proportion ≥ Yes).*

## A Technical details of model and fitting

### A.1 Background to the model

#### A.1.1 Modelling the data via ordinal regression

In our survey, the responses provided have a natural ordering — an individual who responds that they would strongly want to be contacted in the scenario presented to them has a greater desire to know than an individual who responds that they have no opinion. To incorporate this ordered structure we use ordinal regression modelling the probability an individual has, for example, at least a moderate desire to be told. This allows health professionals to determine at what level of desire they perceive action to be justified and then base decisions upon how likely individuals are to be above this level.

#### A.1.2 Inclusion of random effects

Since each individual is presented with four vignettes, the data we see are not entirely independent. Rather the responses given by a single individual are expected to be linked. It is likely that some individuals are more information eager (or averse) than others and these individuals would presumably give responses to all the scenarios that reflected this. In a multi-level framework (Goldstein 1987) each individual would form a level-2 cluster with their four responses being the corresponding level-1 units.

This population variation in eagerness is not something which can be accounted for solely by the measured explanatory variables of, for example, age or sex. Instead, for each individual, we must include a random effect term representing their personal information eagerness (or averseness). For a specific question of interest (e.g. views on proband versus relative rights) this random effect will be common to all that individual’s responses independently of the specific scenario they are presented with. Determining the spread of these random effects allows us to investigate the range of variation in opinion within the overall population.

### A.2 Proportional Odds Logistic Regression with Random Effects

We illustrate our approach using the example of whether an individual wishes to be contacted in the event that a relative has been genetically tested. The possible responses are ordered from 1 to 5 — the lowest level of 1 corresponding to a strong desire not to be contacted and the highest level of 5 a strong desire to be contacted. As such a higher number denotes a stronger desire for the information.

Suppose that this question is to be posed to individual *i* (*i*=1,…,955) in a range of scenarios which vary the nature of the disease information they would receive. Each presented scenario will receive response — an integer value between 1 and 5. We might believe their answer to depend upon both the individual themselves and also the nature of the information that they would receive in the scenario presented. The former will stay fixed whilst the latter will change according to the scenario they are presented with. Let us denote the *M* variables relating to the personal characteristics of individual *i* (for example their age, sex, relationship status, …) by . This individual will be presented with four random vignettes and in each let the *N* variables relating to the specific disease information (for example the seriousness of the disease, risk of development, preventability) be for *j*=1,…,4.

We are interested in how these explanatory variables influence the response provided. To do this, we model the log-odds of a response higher than state *k*

(2)

for $i=1, \ldots, 955$and $j=1, \ldots, 4.$ Here β is a vector of unknown fixed regression parameters that informs us about the effect of personal characteristics (e.g. age, sex) on response, γ describes the effect of disease information (e.g. seriousness, preventability) and is a random effect term which denotes individual *i*’s general information eagerness compared with the general population. The standard deviation of the random effect τ is also unknown. In this model the coefficient (or ) are the log-odds ratio of category *k* or higher when the predictor (or ) increases by one unit conditional on the other predictors and the random effect in the model being fixed.

#### A.2.1 A latent space model view

A common way to interpret the above model is via assumption of an unobservable continuous latent variable *y* that is related to the observed response through a “threshold concept” (see Hedeker and Gibboins (1994) for details). Here the latent variable acts as a proxy for an individual’s strength of opinion on a continuous scale and there is assumed to exist a series of thresholds which determine the response provided for a particular value of *y*. Specifically, the response *R* is seen to take a value *k* if the latent variable *y* falls between the two threshold values and . Each threshold value thus denotes the level of underlying continuous strength of opinion that is required to give a response in a certain category. In this latent space view, we can rewrite our random-effects regression model of Equation (2) for the vignette given to individual *i* in terms of the latent space variable

(3)

where follows a logistic distribution. Based upon our data we can estimate the regression parameters β and γ together with the unknown thresholds and the random effect standard deviation τ.

#### A.2.2 Interpreting the model

To maintain identifiability in the model we must select for the categorical variables a baseline from which to measure the effects. In our case we choose the baseline individual characteristics to be a female between 25–40 years old with a university level education who is not in a relationship or religious; the baseline disease characteristics are a serious and modifiable condition. These will correspond to and and all other scenarios will be measured relative to this. The baseline for both pre- and post-risk are taken to be 0 since they are not categorical.

The latent variable concept is illustrated in Figure 2 where in black we show the density of a logistic random variable centred around 0. If we assume that an individual has a random effect then, for the baseline case of individual and disease characteristics, the latent variable will be drawn from this distribution. The response will then take value *k* if the sampled latent variable lies in .

If we now consider altering either the disease or individual characteristics then this shifts the central location of the logistic from 0 by . Hence if a particular (or ) has a positive value it will mean the individual is likely to give a higher response to that question than a baseline individual while a negative value would correspond to a change whereby an individual is likely to give a lower response than the baseline. Inclusion of the random effect implies that for each individual there will be an additional random mean shift which corresponds to an individual’s propensity to be information eager/averse within the general population.


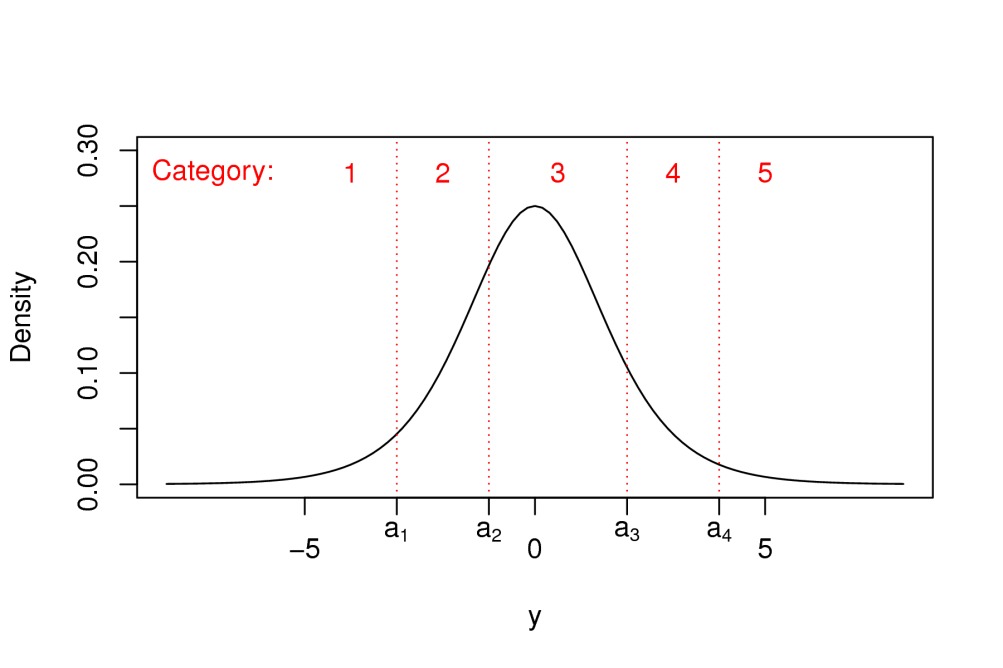


Figure S2: *An illustration of the latent continuous variable. The solid line show the logistic density centred at zero from which a latent variable y will be drawn. This y will fall into one of categories defined by the thresholds which defines the response R. Changing the disease or individual characteristics will shift the density left or right altering the distribution of the latent variable and hence the response too.*

### A.3 A Bayesian Approach

We chose to fit our models in a Bayesian framework using WinBugs (Speigelhalter *et al.* 1997) run within the R software environment (R Core Team 2013). We placed the following uninformative priors on the unknown parameters

$\beta_{l}\sim N\left( 0,1000 \right) \text{for} l=1, \ldots, M\gamma_{l}\sim N\left( 0,1000 \right) \text{for} l=1, \ldots, N$

$\tau^{2}\sim Inv-Gamma\left( 0.001, 0.001 \right) a_{i}\sim U\left[ -15,15 \right] \left( \text{subject to }a_{1}<a_{2}<a_{3}<a_{4} \right)$

For each of the three questions, five over-dispersed chains were run for 20,000 iterations (30,000 in the case of asking whether an individual would forgo their right to confidentiality). The first 10,000 iterations were discarded as burn-in (15,000 for the forgo question) with the remainder used for analysis. Convergence was assessed within the coda package (Plummer *et al.* 2006) using the Brooks-Gelman-Rubin convergence diagnostic (Gelman and Rubin 1992; Brooks and Gelman 1998).

### Supplementary Information References

S. P. Brooks and A. Gelman, “General methods for monitoring convergence of iterative simulations,” *Journal of Computational and Graphical Statistics*, vol. 7, pp. 434–455, 1998.

Gelman and D. B. Rubin, “Inference from iterative simulation using multiple sequences,” *Statistical Science*, vol. 7, pp. 457–511, 1992.

H. Goldstein, *Multilevel Models in Educational and Social Research*. New York: Oxford University Press, 1987.

D. Hedeker and R. D. Gibbons, “A random-effects ordinal regression model for multilevel analysis,” *Biometrics*, vol. 50, no. 4, pp. 933–944, 1994.

M. Plummer, N. Best, K. Cowles, and K. Vines, “CODA: Convergence Diagnosis and Output Analysis for MCMC,” *R News*, vol. 6, no. 1, pp. 7–11, 2006.

R Core Team, *R: A Language and Environment for Statistical Computing*. R Foundation for Statistical Computing, Vienna, Austria, 2013.

D. Spiegelhalter, A. Thomas, N. Best, and D. Lunn, “Winbugs user manual,” 2007. Version 1.4.3.
